# Does Chicago Need Two Medical Societies?

**Published:** 1878-03

**Authors:** 


### Editorial.

---

### DOES CHICAGO NEED TWO MEDICAL SOCIETIES?

Prior to the great fire of October, 1871, there was but one medical association in Chicago—the Chicago Medical Society—and its meetings, in the centre of the city, were so attended that no one would have seriously considered the idea of establishing another with a similar organization, scope and character.

The fire suddenly destroyed the central portion of the city, and then the remaining two sections—the South and West Divisions—were separated by a vast area of débris and ashes. The business and commerce of Chicago having lost their common centre, crystallized around two new nuclei, the one on the West and the other on Twenty-second street, on the South Side. Chicago had, in fact, become divided into two cities. Under these circumstances it was impossible for the Chicago Medical Society to meet at a place which would have suited the convenience of the members of the profession in both towns. The next best plan for the society to adopt would have been to hold alternate meetings in each Division. Although practically this arrangement would have split the society into west and south clubs, unity would have been preserved in name and organization, and no difficulty would have arisen to prevent the reunion of the two in one common meeting as soon as a central location was to be had.

But when, after the October catastrophe, the Chicago Medical Society established itself permanently on the West Side, the physicians residing in the South Division were practically debarred from regularly attending the meetings. They therefore founded a society of their own—the Chicago Society of Physicians and Surgeons.

Under the circumstances this schism was unavoidable, and, justifiable too, if looked upon as a temporary expedient to meet the wants of the profession in the best way possible until the centre of the city could be rebuilt. Continued beyond that period it became a great evil and a calamity to both societies. As each of them in its transactions aims to occupy the whole field of medical science, they are not supplementary to each other as a pathological, or a chirurgical or a gynæcological society would be to a general medical association. They are directly antagonistic because as *town* societies, they prevent a free interchange of opinions among the physicians of the whole city. They do not promote professional friendship but on the contrary alienate the physicians of the several towns ; for very few of the former are able and disposed to attend a weekly meeting. He does very well indeed who attends once every fortnight. Besides there is no particular reason for belonging to two societies so much alike in character. The result is that a physician in one Division will attend the meetings held in his vicinity and thus forfeit the pleasure of seeing and hearing his professional friends elsewhere. The Society of Physicians and Surgeons therefore was very seldom called upon to confer its membership upon West Side practitioners and the Chicago Medical Society very rarely saw the face of those resident in the South and North Divisions, though the names of most members of the first named society appeared in its list of members.

Meetings of medical men in Chicago will never be very largely attended, because of the extent of the district over which residences are scattered. Still one medical society could most assuredly command a fair attendance, if it should receive the patronage of the profession of the whole city. But if this patronage is divided among two societies of the same kind, both institutions will have to suffer, and sooner or later experience the inevitable effect of separation. Already one society has been suffering from chronic absenteeism among its members, and at the other, counting nearly one hundred and fifty members, the attendance has sometimes diminished to little more than a dozen individuals.

West Side physicians have already become so thoroughly accustomed to meeting on their side of the river, that when the Society of Physicians and Surgeons extend an invitation to *all* members of the profession to listen to the reading of an excellent essay on the anatomy of the lymphatic system, the profession of the West Side was represented by but two or three gentlemen.

These observations demonstrate conclusively that there is no need for two similar medical societies in Chicago, and that it is time to close the hiatus which has separated and estranged the members of the profession before it widens and deepens. There is not a single valid apology for the continuance of this anomaly, since the extraordinary circumstances which created it, have ceased to exist.

Let the members of both societies come together, to clasp hands and to unite their efforts in one great aim—to establish one medical society, whose meetings shall become both interesting and attractive by the active rôle assumed by the leading physicians of the city ; whose transactions shall be the pride of the profession of this Western metropolis, and whose membership shall be a high honor to every physician.

